# Online Scene Semantic Understanding Based on Sparsely Correlated Network for AR

**DOI:** 10.3390/s24144756

**Published:** 2024-07-22

**Authors:** Qianqian Wang, Junhao Song, Chenxi Du, Chen Wang

**Affiliations:** The School of Computer and Artificial Intelligence, Beijing Technology and Business University, Beijing 102488, China; chuy0603@163.com (Q.W.); sjhao12@163.com (J.S.); dcx270806@163.com (C.D.)

**Keywords:** real scene analysis, instance segmentation, RGBD SLAM, augmented reality

## Abstract

Real-world understanding serves as a medium that bridges the information world and the physical world, enabling the realization of virtual–real mapping and interaction. However, scene understanding based solely on 2D images faces problems such as a lack of geometric information and limited robustness against occlusion. The depth sensor brings new opportunities, but there are still challenges in fusing depth with geometric and semantic priors. To address these concerns, our method considers the repeatability of video stream data and the sparsity of newly generated data. We introduce a sparsely correlated network architecture (SCN) designed explicitly for online RGBD instance segmentation. Additionally, we leverage the power of object-level RGB-D SLAM systems, thereby transcending the limitations of conventional approaches that solely emphasize geometry or semantics. We establish correlation over time and leverage this correlation to develop rules and generate sparse data. We thoroughly evaluate the system’s performance on the NYU Depth V2 and ScanNet V2 datasets, demonstrating that incorporating frame-to-frame correlation leads to significantly improved accuracy and consistency in instance segmentation compared to existing state-of-the-art alternatives. Moreover, using sparse data reduces data complexity while ensuring the real-time requirement of 18 fps. Furthermore, by utilizing prior knowledge of object layout understanding, we showcase a promising application of augmented reality, showcasing its potential and practicality.

## 1. Introduction

The deep integration of technologies such as computer simulation and augmented reality with manufacturing has accelerated the development of intelligence and precision in the consumer electronics manufacturing industry, leading to a surge in digital twin technology. The core of intelligent manufacturing is to achieve interaction and integration between the digital world and the physical world [1,2]. It demands a precise perception of real-world geometric and semantic information, enabling the extension of new functionalities to physical entities through data parsing, virtual simulation, and virtual–real interaction. This integration empowers intelligent manufacturing to operate with enhanced efficiency and effectiveness. Therefore, building a real 3D scene semantic model is the primary task in establishing a high-density and real-time connection between the information and physical worlds. First and foremost, it is essential to achieve the reconstruction of large-scale and dense geometry to simulate the real world. Specifically, a comprehensive scene-understanding capability to discern individual objects becomes crucial for facilitating reality–virtual interaction. The most prevalent approach involves utilizing RGB sensors to perceive the 3D world through image sequences and processing the data stream into a digitized representation. This process entails reconstructing a dense geometric model, identifying objects of interest within a scene, and categorizing these objects into specific categories. Although RGB images are commonly used for analyzing scene content, their ability to provide comprehensive and accurate analyses is limited by the absence of stereo information. To overcome this limitation, there has been significant attention towards indoor scene understanding based on RGBD images [3,4]. The comprehensive analysis must understand the geometric structure and the object layout relationship.

Several simultaneous localization and mapping (SLAM) methods have been proposed to leverage depth sensors, like Microsoft Kinect for camera motion estimation and dense geometric model reconstruction. Methods such as KinectFusion [5], BundleFusion [6], and OcclusionFusion [7] have achieved real-time performance. Significant progress has been made in 2D semantic analysis tasks like object detection [8] and instance segmentation [9], contributing to developing a knowledge model. However, these remarkable achievements have primarily focused on geometric or semantic understanding separately. In other words, the reconstructed geometric models lack semantic information, and the semantic labels are limited to individual RGBD frames.

To reconstruct a 3D scene model with geometric and object-level semantic information, we propose a novel approach combining instance segmentation with SLAM, resulting in an object-level RGBD SLAM system. The current semantic-level SLAM systems primarily generate sparse maps. The main improvements have been focused on three aspects: utilizing semantic information for real-time performance [7], enhancing the accuracy of camera localization [9], and ensuring data stability during the reconstruction process in large-scale scenes [10]. However, the main challenge lies in designing a real-time instance segmentation network that can be seamlessly integrated with the SLAM algorithm. In particular, existing semantic instance segmentation methods often fall short when applied independently to single images without contextual information. Since consecutive frames in a video stream typically exhibit small camera motion amplitudes resulting in highly similar images, it is natural to leverage this similarity to relate previous segmentation results to the current frame. Additionally, the distribution of different objects within a scene tends to be sparse. Inspired by the characteristics of sparse convolution, we investigate an enhanced sparse convolution technique for efficient instance labeling.

Our work introduces a real-time instance-level RGBD SLAM system based on sparse convolution. Our system captures a sequence of RGBD input frames, such as those obtained from a Kinect sensor. It performs the following tasks: computing 6-degrees of freedom (6DoF) rigid poses, labeling object instances, and reconstructing 3D semantic instance models. To incorporate contextual relevance, we propose a deep network architecture called the Sparsely Correlated Network (SCN) for labeling object instances in each RGBD frame. Firstly, we employ a series of 2D convolutions to extract texture and geometric features from RGBD images. Additionally, we leverage the projection correspondence to establish feature associations across time. For data spaces that lack contextual relevance, we record their coordinates following a predefined rulebook, resulting in sparse data representation. Next, we employ spatially sparse convolution to extract features and use a region proposal network to infer the bounding box locations and semantic labels for object instances. Finally, we fuse the consecutive instance segmentations into a consistent instance map.

To sum up, our contributions are the following:

- We introduce a novel neural network architecture called SCN, which utilizes sparse convolution for online instance segmentation. Combining the instance segmentation network with an RGB-D SLAM system achieves scene understanding at the object level.
- We recognize projective associations between contexts and use hash tables to organize sparse data without association. We also design a sparse convolution architecture to process data without context relevance, which satisfies the real-time requirement.
- We devise a sparse rule for sparse convolution and develop a grouping strategy for dense data by leveraging spatial topological structures and temporal correlation properties.

## 2. Related Work

**Dense RGB-D SLAM.**

The Visual SLAM has a long-standing history of tracking camera motion and reconstructing sparse model maps. The emergence of consumer-grade depth cameras has advanced research in this area, enabling dense and real-time scene mapping. Recent methods have demonstrated accurate mapping of indoor environments, which in turn has addressed the major requirements in fields such as industrial production and intelligent manufacturing. These advancements have paved the way for further developments and applications in these domains. KinectFusion [5] harnesses the advantages of the truncated signed distance function (TSDF) and regularized voxel grid representation to achieve real-time camera tracking and robust scene mapping. To overcome the limitations of space representation, subsequent studies [6,11] extended these principles to large-scale environments by utilizing data structures such as hash tables. Furthermore, an alternative representation based on surfels [12,13] has been proposed to approximate sparse point clouds, where attributes such as normal and radius encode associations between points and surfaces, thereby reducing data redundancy and optimizing data space utilization.

**Instance Segmentation**. 

In recent years, convolutional neural network architectures have significantly advanced object detection [14] and instance segmentation [15]. These advancements have greatly improved the accuracy and efficiency of these tasks, pushing the boundaries of computer vision research.

Early instance segmentation methods mainly concentrated on single RGB images and can be classified into two categories: two-stage methods [16] and one-stage methods [17]. The two-stage methods generally achieve higher segmentation accuracy, while the one-stage methods pursue real-time performance. However, RGB-based instance segmentation methods often suffer from severe error segmentation in low-contrast scenes, as the texture information struggles to reflect the geometric topology between object instances. With the advent of consumer-grade depth sensors, integrating depth information with color and texture has been demonstrated to improve semantic segmentation accuracy [18,19]. In addition, instance segmentation approaches targeting 3D models can provide more comprehensive global information in practical applications. Voxel-based methods represent voxel data in a format suitable for neural network processing. For instance, VoxNet [20] applies convolutions and pooling operations in three dimensions to capture spatial features within 3D data. Voxelization [21] transforms 3D voxel data into a fixed-size voxel grid, making it compatible with input to neural networks. Inspired by PointNet [22], point clouds have gained significant attention due to their advantage of low model representation complexity [23,24]. RandLA-Net [23] proposed a local feature aggregation module to capture the relative positional relationships between points in a local neighborhood, enhancing the efficiency of point cloud recognition by establishing associations between central points and neighboring points. On the other hand, KPConv [24] introduces a variable point convolution that calculates the kernel transformation matrix using an arbitrary number of kernel points, thereby extracting local compelling features in the spatial domain of the point cloud. These point-based methods leverage the spatial information inherent in the point cloud data to perform instance segmentation tasks.

From the perspective of candidate box generation, instance segmentation methods can be categorized into anchor-based detection and anchor-free grouping methods. Anchor-based detection utilizes a predefined set of anchors to generate a proposal and perform classification and pixel-level segmentation for each candidate box. Faster R-CNN [25] proposes a region proposal network (RPN) to generate candidate boxes, speeding up the region proposal process by sharing convolutional layers. Integrating feature extraction, candidate box selection, classification, and bounding box regression into a single end-to-end network effectively enhances detection accuracy and efficiency. He et al. [26] extend Faster R-CNN with a segmentation branch, forming Mask R-CNN, which generates masks for pixels within each candidate box, enabling simultaneous object detection and instance segmentation, thus enhancing segmentation accuracy and precision. Sparse R-CNN [27] replaces the dense anchors in the RPN network with a fixed set of candidate regions, enabling a transition from dense to sparse representation. In contrast to anchor-based detection, anchor-free methods do not rely on predefined anchors but directly generate bounding boxes and masks from the image. Jiang et al. [28] propose a bottom-up 3D instance segmentation framework based on point cloud data, assigning points in the point cloud to different object instances or semantic categories using adaptive neighborhood clustering and global optimization, effectively handling 3D instance segmentation tasks. Zhong et al. [29] introduce a hierarchical point grouping algorithm called MaskGroup. They also present a novel MaskScoreNet to generate binary point masks for all grouped instances, eliminating noisy points. Additionally, MaskScoreNet predicts confidence scores for the final instances to refine the segmentation results further.

**Semantic RGBD SLAM**. 

From the semantics perspective, previously proposed semantic mapping systems can be categorized into two main approaches: the dense labeling and the object-level, as shown in Table 1. SemanticFusion [30] combines the VGG16 network architecture to achieve pixel-level semantic segmentation. Additionally, it updates the class probability distribution using a Bayesian model, enhancing the accuracy and reliability of the segmentation results. DA-RNN [31] introduces data-associated RNNs to establish spatiotemporal associations, achieving more accurate and consistent results through global feature preservation. However, these approaches primarily address scene-level semantic labeling and do not explicitly address the localization and distinction of individual objects within the scene. SLAM++ [32] is a real-time object-level incremental SLAM system. This method constructs an object model library and continuously detects the actual object in the model library to feed into a pose graph. Tateno et al.’s approach [33] is used for rigid body instance segmentation but requires a prior model. These previous methods focus on object-level analysis but limit the categories of objects to the 3D object database. MaskFusion [34], Fusion++ [35], and MID-Fusion [36] have taken advantage of the advantages of using instance-level semantic segmentation and building object-oriented map representation. MaskFusion [34] goes beyond the traditional system of outputting only geometric maps and integrates multiple tasks such as identifying, partitioning, and assigning semantic classes to different moving objects. However, the quantitative detection accuracy is not outstanding because it mainly concerns the camera trajectory accuracy. PanopticFusion [37] achieves comprehensive 3D geometrical and semantic volumetric mapping, enabling the discrimination of individual objects based on Mask R-CNN [26]. Additionally, it applies a fully connected conditional random field (CRF) model to regularize the map by panoptic labels. This regularization step enhances the consistency and coherence of the resulting map, further improving the accuracy of object discrimination and semantic understanding. However, the time efficiency cannot meet the real-time requirement because it takes 235 ms in the object detection network stage. By integrating a two-stream object detection network with a SLAM system, Li et al. [38] achieved dense construction and specific objects while maintaining a sparse global map. This approach successfully improves memory requirements and computational complexity. However, global sparsity mapping limits its potential applications in digital twins. In contrast to the studies mentioned above, our method mainly considers the time requirement of the system, recognizes the context data association relation of the video stream data, and reconstructs a 3D semantic model with global consistency.

## 3. Method

**Figure 1:**
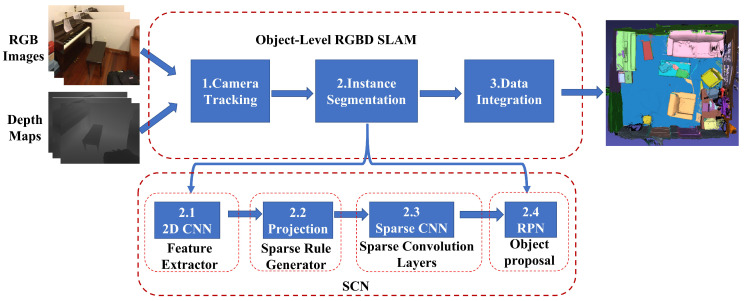
Overview of our object-level RGBD SLAM based on SCN. In three steps, the input RGBD data stream is used to achieve 3D semantic model reconstruction. Step 1: frame-by-frame camera localization is performed. Step 2: camera positions and image data are fed into the SCN network to obtain per-pixel object semantic information. Step 3: the current-time semantics are transformed into global semantics.

The system framework of our approach is illustrated in Figure 1. Building upon the RGBD reconstruction method [15], we incorporate an instance segmentation module into our pipeline. Our approach begins by estimating the camera motion and computing the 6DOF pose (Section 3.1). Subsequently, we input the current RGBD frame into SCN networks to obtain pixel-wise object instance labels, fused to generate accurate segmentation results (Section 3.2). To ensure robust tracking, the instance labels are carefully referenced against the volumetric map at that particular moment. Furthermore, we integrate the probability distributions of class labels into the map, incorporating depth measurements for improved accuracy (Section 3.3).

### 3.1. Camera Tracking

To obtain the corresponding vertex map at time *t*, we transform the raw depth image $D_{t}$ using the camera intrinsic parameters K, resulting in $V_{t}\left(u\right)=D_{t}K^{-1}\in R^{3}$. Additionally, we calculate a normal map $N_{t}$ using the method proposed in [7]. To estimate the six-degrees of freedom camera pose T=[R|t], which includes rotation matrix $R\in {\mathrm{SO}}_{3}$ and translation vector $t\in R^{3}$, we utilize an iterative closest point algorithm similar to the one described in [39]. This algorithm iteratively refines the pose estimation by aligning the observed 3D points with the reconstructed model.

### 3.2. Instance Segmentation

After estimating the camera’s motion, we need to predict pixel-wise object instance labels. Our network architecture is also shown in Figure 1. It consists of four primary components: a feature extractor (Section 3.2.1) for transforming raw RGB and depth images into a more meaningful and representative format, sparse rule generator (Section 3.2.2) for associating data with geometric consistency (i.e., data that the camera have already observed) and recording newly observed data, sparse convolution layers (Section 3.2.3) for learning the feature description of newly generated data, and object proposal (Section 3.2.4) like object detection for identifying the object instance and predicting semantic class. In the following, we will outline the critical components of our architecture design.

#### 3.2.1. Feature Extractor

For the initial frame at time t=0, the detection accuracy plays a critical role in the subsequent online learning process. To achieve this, we utilize the Mask R-CNN [26] to generate pixel-level segmentation results, including the object category $L_{1}\left(u\right)\in L$; the object instance ID $Z_{1}\left(u\right)\in Ƶ$; the surrounding bounding box $B_{1}=\left\{<▵x_{1}^{1},▵y_{1}^{1},w_{1}^{1},h_{1}^{1}>,...,<▵x_{num_{o}}^{1},▵y_{num_{o}}^{1},w_{num_{o}}^{1},h_{num_{o}}^{1}>\right\}$ representing the coordinates of each bounding box, respectively; and the feature map $f_{1}^{rgbd}$, obtained by fusing the RGB and depth modalities resulting in a comprehensive representation that encompasses both visual and geometrical information. In the context of our method, $L=\{l_{1},...,l_{num_{l}}\}$ represents a collection of all possible object types in the indoor scene, where $l_{1}$ to $l_{num_{l}}$ denote the individual object types. The $num_{l}$ represents the total number of object species. The $Ƶ=\{1,...,num_{o}\}$ represents a set of object IDs, ranging from 1 to $num_{o}$, where $num_{o}$ corresponds to the total number of objects. The u=(x,y) denotes the pixel location. Overall, Mask R-CNN enables us to obtain accurate object segmentation results, including object categories, instance IDs, bounding boxes, and fused feature mappings. These results are the foundation for subsequent online learning and further analysis of our method.

**Figure 2:**
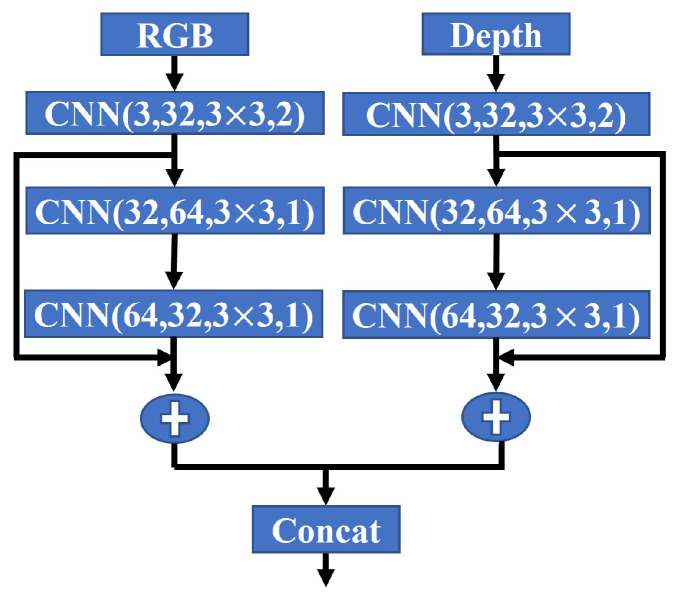
ResNet blocks for 2D feature learning.

We employ the ResNet architecture for the subsequent frames to generate initial 2D feature maps. In Figure 2, the 2D network architecture separately takes the H×W RGB and depth image as inputs. We extract multi-modality features: $f_{n}^{r}$ (H×W×c) from the RGB encoder and $f_{n}^{d}$ (H×W×c) from the depth encoder. These feature maps capture meaningful representations of the input data. To fuse these features, we use a concatenation layer, which combines the feature maps into a single input ${\hat{f}}_{n}^{rgbd}$ ($H\times W\times c_{in}$) for the sparse convolution layers. This input retains the spatial dimensions (H×W) but has a different number of channels ($c_{in}$) determined by the concatenation of the RGB and depth features.

#### 3.2.2. Sparse Rule Generator

**Figure 3:**
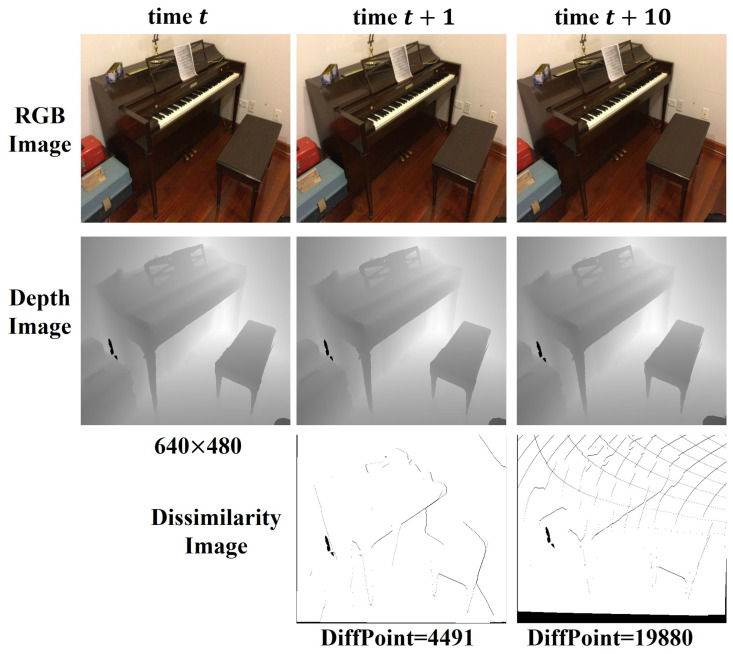
Image differences at different time intervals.

The composite features need to correlate the feature information in the previous part through the projection and organize sparse data for unrelated data. Minimal camera motion at adjacent moments leads to high similarity in the image, as shown in Figure 3. At times *t* and t+1, the new data collected by the camera only account for about 15 percent of the entire image and are distributed discreetly. Therefore, it is unnecessary to calculate the image feature description repeatedly for data with geometric consistency. We project the current coordinates back to the previous time to establish the temporal connection relationship. For each pixel position $u_{t}$ and the corresponding vertex map $V_{t}\left(u\right)$, we calculate the world coordinates $W_{t}\left(V_{t}\left(u\right)\right)$ based on the current camera pose. Next, we aim to find the projection location $u_{t-1}^{*}$ at the previous time step, i.e., $W_{t-1}\left(V_{t-1}\left(u^{*}\right)\right)\approx W_{t}\left(V_{t}\left(u\right)\right)$. We can associate the feature between the two consecutive frames by establishing this projection relation.

**Figure 4:**
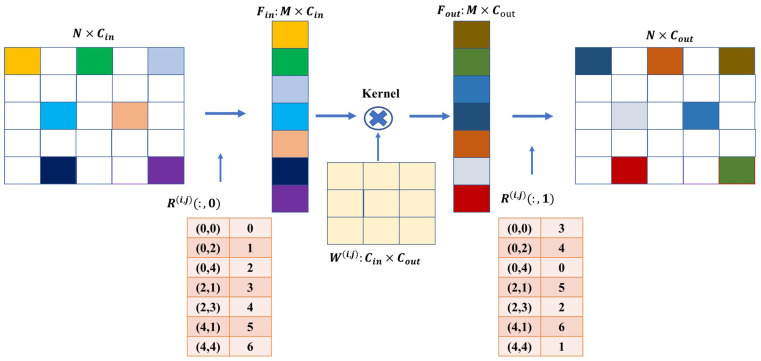
Specific rules for sparse data convolution.

Complete convolution operations are not appropriate when dealing with data that lack inherent correlation due to their discrete distribution. We can employ sparse convolution, as described in [40], for deep feature learning. This approach requires designing rules to organize discrete data as sparse, as depicted in Figure 4. The size of the input data is denoted as $N\times C_{in}$, where $N=h_{in}\times w_{in}$ represents the field size and $C_{in}$ denotes the number of feature map channels. The filter kernel size is given by ($C_{in},C_{out},k\times k,s$), where *k* denotes the size of the convolution kernel and *s* represents the stride. We apply this filter to the input data results with an output data size of $C_{out}\times h_{out}\times w_{out}$, with $h_{out}=\left(h_{in}-k\right)/s$ and $w_{out}=\left(w_{in}-k\right)/s$.

We utilize a hash table structure to organize the input data as sparse data. This hash table is responsible for recording the positions of the sparse data during the convolution process. More specifically, the input data $F_{in}$ have a size of $M\times c_{in}$ and only retain information about the sparse data. Each row vector in $F_{in}$ represents the feature vector of *M* sparse pixels across all $c_{in}$ channels. Furthermore, we introduce another hash table called *R*, which records the location map of the sparse pixels in the raw input data as {(x,y),value}. Here, (x,y) indicates the pixel position and value is the row index in $F_{in}$. The maximum size of the hash table *R* is $h_{in}\times w_{in}$, which typically occurs during the initial object detection phase when there are the most sparse pixels in the data. We multiply the weight matrix parameter *W* with $F_{in}$, and the output feature map $F_{out}$ has a size of $M\times c_{out}$. The weight matrix *W* has dimensions $c_{in}\times f\times f\times c_{out}$, and $W^{\left(i,j\right)}$ has a size of $c_{in}\times c_{out}$, where (i,j) represents the offset to the center of the convolution kernel. For a specific offset (i,j), the input feature matrix can be fetched using $R^{\left(i,j\right)}\left(:,0\right)$. After multiplying it by the corresponding weight parameters $W^{\left(i,j\right)}$, the output data are scattered and constructed as the output feature matrix according to the output pixel index in $R^{\left(i,j\right)}\left(:,1\right)$. In summary, we can effectively address the challenges associated with data lacking inherent correlation by incorporating sparse convolution techniques and designing rules to organize discrete data.

#### 3.2.3. Sparse Convolution Layers

**Figure 5:**
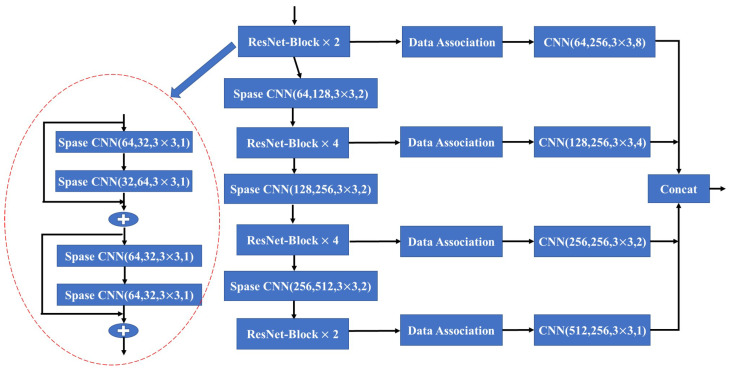
The detailed architecture of Sparse Correlated Network.

Following the rules mentioned above, the sparse convolution layers leverage the fused input to learn and extract relevant features. These layers operate sparsely, focusing on specific regions of interest rather than processing the entire input space. This approach allows the network to effectively capture and encode the most informative aspects of the data while minimizing computational overhead. Building upon these sparse convolution rules, we construct the pyramidal feature hierarchy network named Sparse Correlated Network (SCN), as shown in Figure 5.

We establish connections between the four pyramidal levels to produce high-level feature maps, which use convolutional layers with stride sizes of (8, 8), (4, 4), or (2, 2) to capture multi-scale feature details. Combining the associated features from the previous frame with the current sparse features after performing a series of sparse convolutions is crucial to ensure a complete feature map. Specifically, we partition the dense feature maps into two datasets, denoted as O and N. Let us assume that $u_{n}=\left(x_{n},\;y_{n}\right)$ represents the pixel position index in the $n_{th}$ frame’s depth image $D_{n}$. Using the camera extrinsic $T_{n}$, we can obtain the coordinates $W_{n}\left(u_{n}\right)$ at the world coordinate system for the point $u_{n}$. If in the ${\left(n-1\right)}_{th}$ frame’s depth image there exists a point $W_{n-1}\left(u_{n-1}\right)$ such that it is equal to $W_{n}\left(u_{n}\right)$, then we update the O set as follows: $O=O\cap f_{n-1}^{rgbd}\left(u_{n-1}\right)$. Otherwise, we update the N set: $N=N\cap {\hat{f}}_{n}^{rgbd}\left(u_{n}\right)$. *N* is the SCN input and accomplishes local feature extraction by employing sparse convolution. These extracted features are subsequently processed through the data association module, creating a dense feature map. Ultimately, the feature map is outputted using a concatenation layer and represented as $f_{n}^{rgbd}$.

#### 3.2.4. Object Proposal

To optimize computational efficiency, we introduce several steps in our approach. Firstly, we transform the center coordinates $\left(▵x_{i}^{n-1},▵y_{i}^{n-1}\right)$ of each bounding box in the set $B_{n-1}$ to the world coordinate system and project them onto the corresponding positions $\left(▵x_{i}^{n},▵y_{i}^{n}\right)$ in the $n_{th}$ frame. Compared to the anchor mechanism with no prior knowledge, we initialize the bounding box set as $B_{n}^{*}=\left\{b_{1},...,b_{i},...,\right\}$, where each $b_{i}$ is represented as $b_{i}=<▵x_{i}^{n},▵y_{i}^{n},w_{i}^{n-1},h_{i}^{n-1}>$. Next, we compute the Intersection over Union (IoU) between each $b_{i}$ in $B_{n}^{*}$ and each $a_{j}$ in the anchor set A. This calculation is performed using the formula $IoU\left(b_{i},a_{j}\right)=|b_{i}\cap a_{j}\left|/\right|b_{i}\cup a_{j}|$. If the resulting IoU value exceeds a predefined threshold $\theta_{U}$, denoting significant overlap, we remove the corresponding anchor $a_{j}$ from the anchor set A. Finally, we employ a bounding box regressor to obtain comprehensive object instance information that refines the remaining anchors in A. This regression process helps refine the localization and characteristics of the detected objects. By incorporating these optimization techniques, our method effectively reduces the computational burden and improves the overall efficiency of the system while accurately identifying object instances.

### 3.3. Data Integration

Semantic reconstruction involves continuously updating and fusing depth image data, known as incremental modeling [39]. Each captured depth image frame is fused into a globally consistent 3D voxel-based model according to the camera pose. The TSDF measures the distance between each voxel grid in the model and the model representation, ensuring geometric consistency constraints. Additionally, semantic elements such as class probabilities and instance bounding boxes are incorporated into the voxel grid to ensure semantic consistency. Through these steps, a 3D model is generated that satisfies both geometric and semantic consistency.

## 4. Evaluation

### 4.1. Dataset

The proposed methods are validated using the ScanNet v2 dataset [41]. This large-scale RGB-D dataset is used for indoor scene understanding and reconstruction. It primarily consists of diverse indoor scenes such as offices, homes, stores, and schools. The dataset comprises 1201 training scenes and 312 open test scenes. For each scene, it provides camera poses, semantic labels, instance labels, and surface normals. For testing purposes, we also utilize the NYU Depth v2 dataset [42], which includes 1449 RGBD images covering 40 common semantic types.

### 4.2. Network Details

We utilize the ResNet architecture to process the 640 × 480 RGB image and depth map separately. These inputs are passed through the network, resulting in two features of dimensions 320×240×32. The features are then stacked, resulting in a 64-channel feature. For optimization, we employ the ADAM optimizer with a momentum of 0.9, a learning rate of 0.0002, a batch size of 5, and a sequence length of 10. Our network trains on an NVIDIA GeForce RTX 3090 GPU, taking approximately two days to complete 1800 epochs.

### 4.3. Quantitative and Qualitative Results

To evaluate the effectiveness of SCN, we measure its segmentation accuracy using the mean Average Precision (mAP) of bounding boxes on two datasets: NYU Depth v2 [42] and ScanNet v2 [41]. The performance of SCN on the NYU Depth v2 is presented in Table 2. Our method demonstrates superior performance, primarily due to Mask R-CNN’s inability to consider the correlation between detection results from consecutive frames in the scene. This confirms the validity of our bounding box generation strategy proposed in the object proposal module, which initializes the t=n frame using the bounding box from the t=n−1 frame.

**Figure 6:**
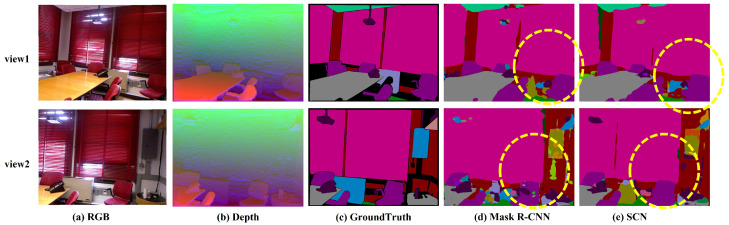
Comparison of semantic segmentation accuracy in NYU depth dataset.

To evaluate the influence of inter-frame correlations on segmentation accuracy, we select two sets of data with different perspectives of the same scene, as seen from Figure 6. Both Mask R-CNN and SCN accurately segment the chair enclosed by the yellow box in view 1. However, there is a discrepancy in the segmentation results of these two methods in view 2. Mask R-CNN makes it difficult to retain the compelling features extracted in the segmentation process from the previous perspective, resulting in incorrect segmentation, which verifies the necessity of SCN to improve the accuracy of instance segmentation.

**Figure 7:**
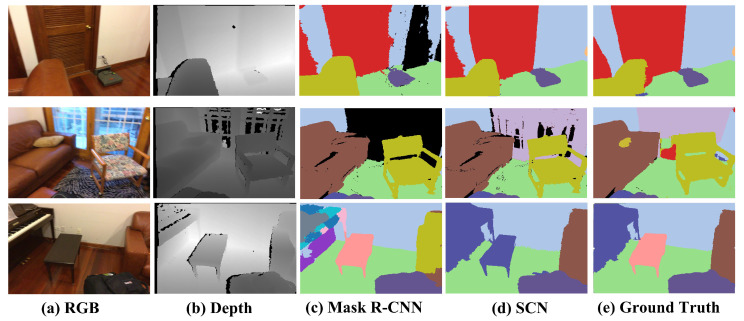
Comparison of semantic segmentation accuracy in ScanNet dataset.

**Figure 8:**
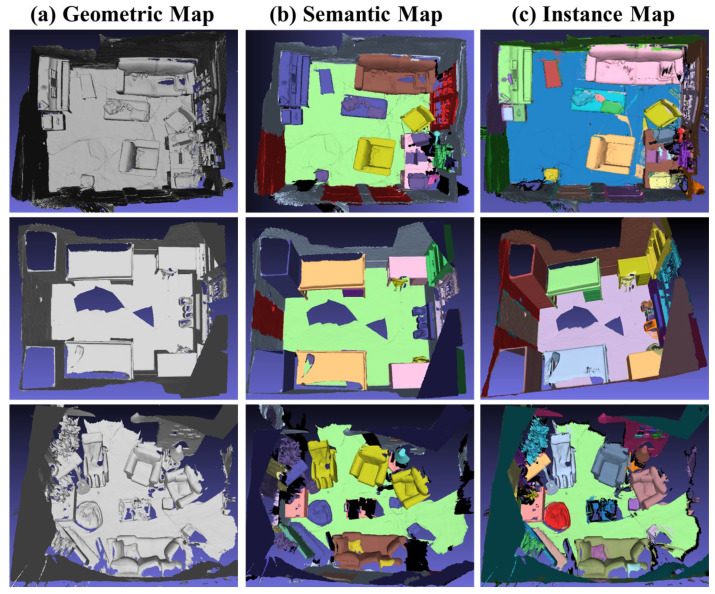
Instance Segmentation results obtained with our network. The integrated geometric model, semantic map, and instance map are displayed from left to right.

We have also applied our SCN method to the ScanNet v2 dataset [41], yielding instance segmentation results. Figure 7 demonstrates the semantic segmentation outcomes of an individual frame image, illustrating the improved accuracy achieved by our method compared to Mask R-CNN [26]. Our results exhibit enhanced correctness in semantic segmentation. Moreover, to establish the global effectiveness of our approach, we have fused the segmentation results from various scenarios into semantic and instance-level 3D models, as depicted in Figure 8. This fusion serves as evidence of the semantic consistency attained by our approach.

**Figure 9:**
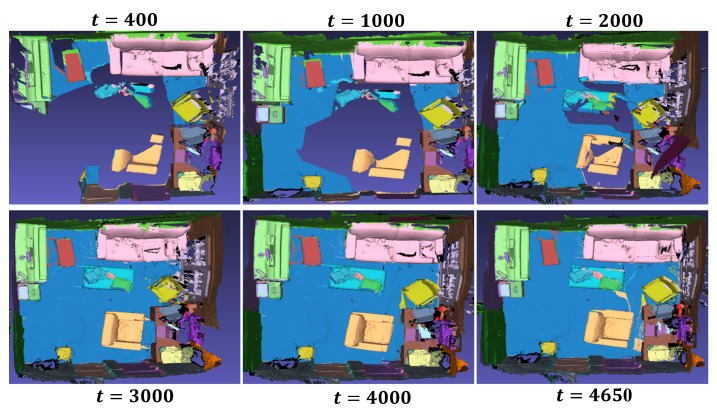
Online 3D semantic model generation based on camera motion.

For quantitative evaluation, we compare our results with per-voxel class accuracies, following the evaluation metrics used in previous works such as Mask R-CNN [26], PanopticFusion [37], Sparse R-CNN [27], PointGroup [28], and Mask-Group [29]. Table 3 numerically compares the mean average precision with the IoU threshold of 0.5 over 19 classes of our method against other deep learning approaches. For instance segmentation, PanopticFusion utilizes Mask R-CNN as the baseline network, Sparse R-CNN adopts sparse convolution techniques, and PointGroup and Mask Group employ point cloud grouping. These methods share similarities in design with SCN. The results highlight that SCN has an advantage in segmenting objects that frequently repeat across multiple frames, such as beds and desks. However, due to the low repeatability of small objects in adjacent frames, the proposed approach for associating features between consecutive frames may not perform optimally, resulting in suboptimal accuracy. Furthermore, Figure 9 demonstrates the object detection process SCN performs in a real indoor scene composed of 4650 depth image sequences.

### 4.4. Run-Time Analysis

Utilizing the parallel computing capabilities of CUDA 10.0 architecture, our RGBD SLAM system achieves an impressive processing time of approximately 55 ms per frame, which includes 5 ms for camera tracking, 30 ms for instance segmentation, and 20 ms for data integration. In general, our indoor scene analysis system achieved a performance of 18 frames per second (fps), allowing for interactive rates of real-time processing. Table 4 compares the running time for object detection using SCN against the depth image sequence in the ScanNet dataset. Our SCN method follows the two-stage detection idea of Mask R-CNN but establishes spatiotemporal correlation within scenes, resulting in shorter running time and higher detection accuracy. In comparison, PanopticFusion directly utilizes Mask R-CNN for object detection in multi-frame depth images and then employs a CRF model for global consistency of object detection results. Compared to PanopticFusion, our method achieves improved detection accuracy and time performance.

### 4.5. AR Application

**Figure 10:**
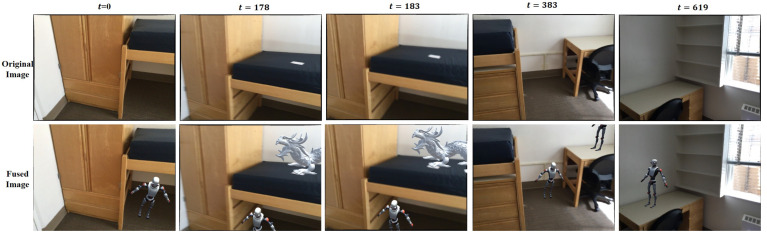
AR applications that combine 3D instance mapping with virtual objects.

By leveraging existing semantic information, we can develop immersive augmented reality applications. Our approach utilizes the spatial distribution of detected objects within the scene, allowing us to seamlessly integrate virtual objects, such as robots and dinosaurs, into the real environment. As shown in Figure 10, this fusion process ensures that virtual objects are rendered realistically, seamlessly blending with the surrounding real scene.

**Figure 11:**
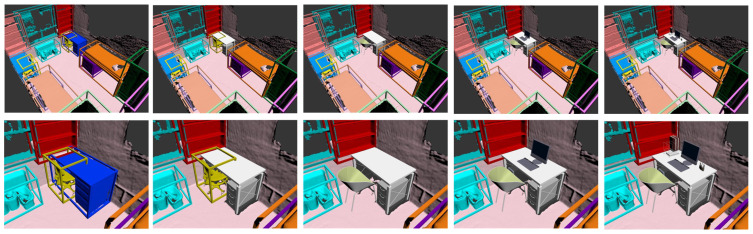
Virtual–real substitution based on semantic association rules.

Furthermore, our system enables the replacement of semantically known objects in the scene, such as desks and chairs, with corresponding semantic 3D models. Additionally, we can enrich the scene presentation by adding semantically associated objects, such as computers, to enhance the virtual reality experience further. Figure 11 demonstrates this augmentation and enrichment of the scene. By incorporating these advancements, our method can provide users with a highly realistic and interactive experience where virtual and real elements coexist seamlessly. The combination of precise spatial alignment and semantically meaningful object placement creates a truly immersive and captivating augmented reality environment and introduces a crucial interface for interactive intelligent manufacturing. This interface aims to redefine the production process. By seamlessly integrating virtual operation commands with real-world industrial production floors, we provide essential technological support for the digital twin platform in intelligent manufacturing, propelling the manufacturing industry into a completely new paradigm.

## 5. Conclusions

We present a new instance segmentation network called SCN, which incorporates an object-level RGBD SLAM system. Our approach recognizes the significance of context correlation in establishing relationships and distinguishing individual objects. In the initial frame, we employ Mask R-CNN to generate the instance segmentation result and establish the mapping rule by leveraging inter-frame correlation during subsequent camera movement. We adopt various feature generation methods to accommodate different data attributes and effectively reduce computational complexity by integrating sparse convolution. Experimental results demonstrate that our network outperforms or achieves comparable performance to state-of-the-art methods regarding instance segmentation. Furthermore, our network meets the required time performance criteria. In future work, we will research dynamic industrial scenarios to enable intelligent analysis and facilitate virtual reality interactions within large-scale complex scenes.

## Figures and Tables

**Table 1:** Semantic mapping methods comparison.

Method	Online	TSDF Volume	Surfels	Large-Scale	Dense Labeling	Object-Level
SemanticFusion [30]	√		√	√	√	
DA-RNN [31]	√	√			√	
SLAM++ [32]	√					√
Tateno et al. [33]	√	√				√
MaskFusion [34]	√		√			√
Fusion++ [35]	√	√				√
MID-Fusion [36]	√	√			√	√
PanopticFusion [37]	√	√		√	√	√
Li et al. [38]	√		√	√		

**Table 2:** Comparison of semantic segmentation results between SCN and Mask R-CNN on NYU depth v2 dataset.

Method	Input	Baseline	mAP (%)
Mask R-CNN [26]	RGB + Depth	VGG	56.9
Mask R-CNN [26]	RGB + Depth	ResNet-101	62.5
SCN	RGB + Depth	VGG	63.4
SCN	RGB + Depth	ResNet-101	71.2

**Table 3:** Comparison of the $mAP_{50}$ of our method against other referential approaches on ScanNet v2 dataset.

Category	Mask R-CNN [26]	PanopticFusion [37]	Sparse R-CNN [27]	PointGroup [28]	Mask-Group [29]	SCN
bathtub	0.333	0.667	**1.000**	**1.000**	**1.000**	**1.000**
bed	0.002	0.712	0.538	0.765	0.822	**0.841**
bookshelf	0.000	0.595	0.282	0.624	**0.764**	0.752
cabinet	0.053	0.259	0.468	0.505	0.616	**0.620**
chair	0.002	0.550	0.790	0.797	**0.815**	0.772
counter	0.002	0.000	**0.173**	0.116	0.139	0.163
curtain	0.021	0.613	0.345	**0.696**	0.694	0.685
desk	0.000	0.175	0.429	0.384	0.597	**0.629**
door	0.045	0.250	0.413	0.441	0.459	**0.462**
otherfurniture	0.024	0.434	0.484	0.559	**0.566**	0.495
picture	0.238	0.437	0.176	0.476	**0.599**	0.424
refrigerator	0.065	0.411	0.595	0.596	0.600	**0.637**
shower curtain	0.000	**0.857**	0.591	1.000	0.516	0.480
sink	0.014	0.485	0.522	0.666	**0.715**	0.692
sofa	0.107	0.591	0.668	0.756	0.819	**0.820**
table	0.020	0.267	0.476	0.556	0.635	**0.661**
toilet	0.110	0.944	0.986	0.997	**1.000**	0.960
window	0.006	0.359	0.327	0.513	0.603	**0.721**
**mAP**	0.058	0.478	0.515	0.636	**0.664**	0.656

**Table 4:** Time comparison.

Method	FPS	Time ms	mAP%
Mask R-CNN [26]	8.6	116.3	5.8
PanopticFusion [37]	4.3	232.5	47.8
SCN	18.0	55.6	65.6

## Data Availability

The data presented in this study are available on request from the corresponding author.
